# Advances in the use of chlorhexidine for periodontitis treatment in diabetic patients: A review

**DOI:** 10.1097/MD.0000000000039627

**Published:** 2024-09-06

**Authors:** Honglan Sun, Shizhao Chen, Chao Yang, Huifang Kuang, Yuqi Huang, Xiaoning He, Wen Luo

**Affiliations:** a Key Laboratory of Emergency and Trauma of Ministry of Education, Department of Stomatology, Key Laboratory of Hainan Trauma and Disaster Rescue, The First Affiliated Hospital of Hainan Medical University, Haikou, Hainan Province, China; b School of Stomatology, Hainan Medical University, Haikou, Hainan Province, China; c Research and Development Department, Shenzhen Uni-medica Technology Co., Ltd, Shenzhen, Guangdong Province, China.

**Keywords:** chlorhexidine, diabetes mellitus, dysbiosis, periodontal therapy, periodontitis

## Abstract

Periodontitis and diabetes mellitus exhibit a bidirectional relationship. This narrative review descriptively outlines the role of chlorhexidine in the periodontal treatment of diabetic patients, focusing on its antimicrobial mechanisms against microbial communities and its antiplaque effects. Although chlorhexidine is proven to be effective in combating microbial presence and improving gingivitis with substantial supporting evidence, its impact on glycemic control and insulin resistance in diabetic patients remains contentious. Additionally, the effectiveness of chlorhexidine as an adjunctive chemotherapeutic in the periodontal treatment of gestational diabetes has not yet been studied, highlighting a gap in research that necessitates further prospective studies and randomized controlled trials. Considering the interconnection between periodontal inflammation and glycemic levels, this article finally advocates for collaborative care between dental and medical professionals to manage periodontitis in diabetic patients effectively.

## 1. Introduction

The global prevalence of diabetes mellitus (DM) among adults has risen to 8.5%, resulting in an annual death toll of 1.5 million.^[1]^ DM encompasses several distinct forms: type 1 diabetes, characterized by the autoimmune destruction of insulin-producing cells; type 2 diabetes, associated with insulin resistance and impaired β-cell function; and gestational diabetes, which develops during pregnancy and generally resolves postpartum. Furthermore, particular variants of diabetes arise due to genetic mutations, exocrine pancreatic dysfunctions, or the influence of specific drugs and chemicals.^[2]^ Patients with diabetes, due to the instability of blood glucose levels, exhibit a spectrum of oral complications, including increased incidence of periodontal disease, xerostomia, fungal infections such as candidiasis, chronic oral ulcers, oral mucosal lesions, alterations in taste perception, gingivitis, heightened risk of dental caries, and an overall reduction in oral healing capacity and susceptibility to infections. As one of the most important complications, periodontitis is a prevalent chronic inflammatory disease characterized by the destruction of tooth-supporting structures. Periodontitis is classified according to the 2017 World Workshop on the classification of periodontal and peri-implant diseases and conditions into four stages (initial to severe) and 3 grades (low, moderate, and high risk),^[3]^ providing a comprehensive assessment of the severity and progression risk of the disease. Previous literatures indicate a significant correlation between DM and periodontitis. DM is linked to increased occurrence and severity of periodontitis. Periodontitis in diabetic patients is a manifestation of the complex interplay between chronic hyperglycemia and oral dysbiosis, where the altered metabolic state impairs immune function and wound healing, thereby fostering an environment conducive to pathogenic microbial communities.^[4]^ In individuals with DM, elevated glucose levels in gingival crevicular fluid can encourage the growth of anaerobic bacteria, altering the composition of the oral microbiota.^[5]^ This dysbiosis, marked by an increase in periodontopathogens such as *Porphyromonas gingivalis* (P.g), leads to a heightened inflammatory response that is both a result of and a contributor to the progression of periodontal disease. The DM, characterized by a reduced host immune response due to impaired neutrophil function and a compromised antioxidant system, fails to maintain the bacterial equilibrium, allowing these pathogens to thrive and cause tissue destruction. Furthermore, advanced glycation end products formed in DM can cross-link with periodontal tissues and matrix components, exacerbating inflammation and tissue breakdown and also providing binding sites that may alter the colonization and virulence of periodontal pathogens.^[6]^

The successful management of periodontitis in patients with DM necessitates a comprehensive approach that encompasses both operative and nonoperative treatments. The basic periodontal treatment is essentially a nonsurgical treatment that eliminates local and systemic pathogenic factors. Improvement of the inflammatory state of the gingiva and the consequent acquisition of pocket depth reduction are the expected goals of basic periodontal therapy.^[7]^ On the part of the patient, it requires a full understanding of the disease and possible prognosis, correction of associated poor performance habits, and improvement of systemic conditions under the guidance of the practitioner.^[8]^ The possible actions of the practitioner include extraction of teeth with no hope of retention, scaling and root planing (SRP), removal of various local plaque retention factors, necessary occlusal interventions, and necessary pharmacological adjuncts. Mechanical debridement in the form of scaling and root planing is considered the gold standard nonsurgical procedure for periodontal therapy.^[9]^ SRP, aimed at removing plaque biofilm, calculus, and endotoxin, face limitations, particularly in deeper periodontal pockets (≥5 mm) where instrument accessibility is restricted.^[10,11]^ Additional antimicrobials are proposed to overcome these problems.^[12]^

Chlorhexidine is a biguanide compound with a structure consisting of two (p-chlorophenyl) guanide units linked by a hexamethylene bridge. It is frequently used in oral health care for its antibacterial properties, commonly as part of mouthwashes, dental gels, and oral sprays. The concentration of chlorhexidine varies depending on the preparation used.^[13]^ Several studies have assessed the positive efficacy of chlorhexidine against periodontal diseases and in reducing plaque accumulation, tooth caries, gingivitis, periodontitis, and alveolar osteitis.^[14–16]^ However, considering the adverse reactions such as staining and allergies associated with chlorhexidine,^[17]^ research interest in alternatives to chlorhexidine has also been reflected in alternative therapies such as probiotics, paraprobiotics, and ozone substances. These alternatives are considered for periodontal therapy due to their potential to balance the oral microbiome and reduce inflammation. Scribante and his team have conducted extensive research in this field. For instance, one of their studies evaluated the anti-inflammatory effects of ozone gel in nonsurgical periodontal disease treatment, finding no significant difference in reducing clinical inflammation markers compared to traditional chlorhexidine gels, which suggests a potential application for ozonated substances in periodontal therapy.^[18]^ Moreover, they have explored the effects of probiotics and paraprobiotics in periodontal therapy, noting that these biological products help improve periodontal health by modulating the oral microbiome and reducing pathogens, thus supporting the treatment of periodontal disease.^[19,20]^

Periodontitis, due to its high prevalence and potential role in the progression of diabetes, imposes a significant global health care burden and presents a worldwide public health challenge. Consequently, there is a need for simple and effective treatment methods to reduce the number of people affected by these two conditions.^[21]^

Mouthwash serves as a noninvasive self-care product that can be easily utilized without disturbing periodontal tissues.^[22]^ The use of chlorhexidine mouthwash is a common oral health care practice, frequently used as an adjunct to mechanical periodontal therapy. The antiplaque efficacy of the mouthwashes is supported by high-level evidence from previous studies.^[23]^ However, evidence supporting the effectiveness of chlorhexidine mouthwash alone for periodontal care in diabetic patients is lacking, particularly among those with gestational diabetes. This review summarizes the mechanisms by which chlorhexidine acts on periodontal tissues and reviews the literature on the effects of chlorhexidine on periodontal parameters, pathogens, glycemic control, and oxidative stress in patients with DM, providing evidence-based guidelines. Additionally, this article actively explores the possibility of a combined approach involving both periodontology and internal medicine for the management of periodontal conditions in diabetic patients.

## 2. Relationship between DM and periodontitis

### 2.1. Mechanisms underlying the bidirectional relationship between periodontal disease and DM

The molecular and cellular mechanisms underlying the association between periodontal disease and DM have been extensively investigated. Shared risk factors such as poor oral hygiene, smoking, and unhealthy diet can contribute to the development and progression of DM and periodontitis.^[24,25]^ The oral microbiome in periodontitis can affect glucose metabolism by influencing the host’s inflammatory response.^[6,26]^ Proinflammatory cytokines released during periodontal disease, such as tumor necrosis factor-alpha and interleukin-6 (IL-6), can increase insulin resistance.^[27,28]^ The chronic inflammation associated with periodontitis can lead to insulin resistance, making it harder for the body to utilize insulin effectively. Additionally, the bacteria present in periodontal pockets can release toxins and proinflammatory cytokines into the bloodstream, further exacerbating insulin resistance. The reduction in levels of proinflammatory cytokines with increasing severity of periodontitis serves as an indicator for successful treatment.^[29]^ The presence of periodontal disease can make it more difficult to control blood sugar levels, leading to a worsening of diabetic conditions. Hyperglycemia leads to the nonenzymatic glycation of proteins, including collagen in the periodontal matrix. Glycated collagen is more prone to degradation and less effective in tissue repair, which undermines the structural integrity of the periodontium.^[30]^ In DM, wound healing is notoriously impaired due to a combination of poor perfusion resulting from microvascular disease, compromised immune response, and altered collagen metabolism, all of which are critical in the maintenance of periodontal tissue integrity.^[31]^

**Figure 1.**
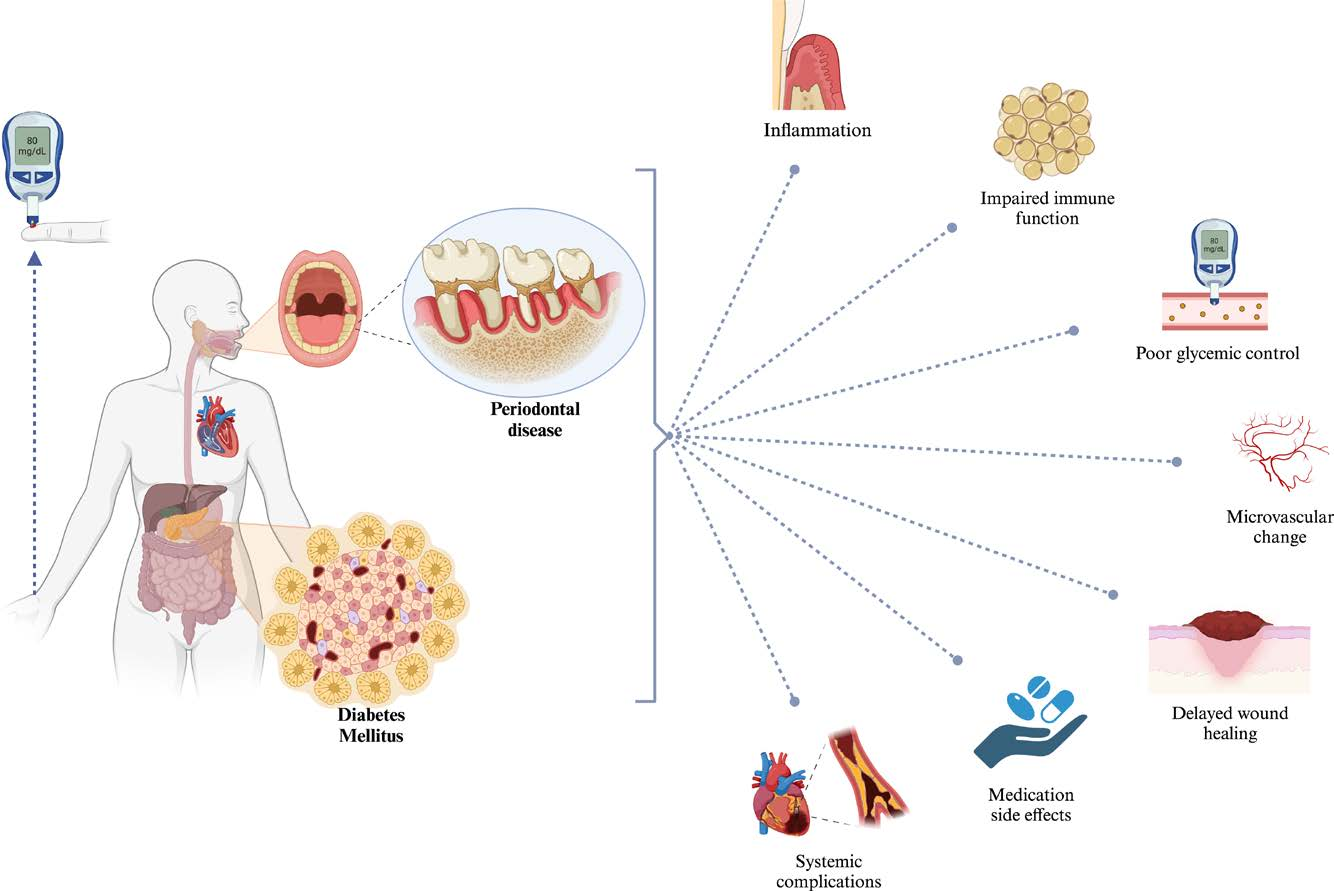
Bidirectional link between diabetes mellitus and periodontitis. The image on the left side depicts a patient with periodontal disease and diabetes. On the right side, the diagram details the interplay between diabetes and periodontal disease, explaining how they contribute to systemic issues. Created with BioRender.com.

DM also impairs neutrophil function, which is crucial for the initial defense against periodontal pathogens. Furthermore, a compromised immune response due to diabetes can result in an inadequate resolution of inflammation.^[32]^ Consistent findings include elevated levels of IL-1β, IL-6, and tumor necrosis factor-alpha in crevicular fluid and saliva; an increased ratio of receptor activator of nuclear factor κB ligand to osteoprotegerin; compromised neutrophil function; the formation of advanced glycation end products; and impaired tissue repair.^[33]^ The healing of periodontal tissues is further impeded by hyperglycemia, which affects both cellular function and matrix remodeling.^[32]^ Cardiovascular disease is another common comorbidity in patients with DM and is similarly associated with periodontitis. The systemic inflammation that characterizes both conditions can contribute to endothelial dysfunction, atherogenesis, and ultimately, cardiovascular events.^[34]^ Periodontal inflammation can exacerbate this risk by contributing to the systemic inflammatory and atherogenic burden.^[35]^ Microvascular complications in DM, characterized by thickening of the vascular basement membrane and endothelial dysfunction, result in reduced blood flow to various tissues, including periodontal tissues.^[36]^ These alterations can exacerbate periodontal disease progression and reduce the efficacy of periodontal treatments.^[37]^ Moreover, the pharmacological management of DM can have side effects that affect periodontal health. For instance, drugs like dipeptidyl peptidase-4 inhibitors have been associated with an increased risk of infections, which could potentially worsen periodontal disease.^[32]^ Conversely, medications used in the treatment of periodontitis may influence blood glucose control, necessitating careful coordination of care between health care providers.^[38]^ Although the precise mechanisms underlying the association between diabetes and periodontitis remain incompletely elucidated, there is a well-established understanding of the involvement of inflammatory cytokines, immune function, glycemic control, microvascular change, wound healing, medication side effects, and systemic complications (shown in Figure 1).

### 2.2. Mechanics of dysbiosis

DM is associated with impaired neutrophil function, which reduces the host’s ability to contain and clear bacterial infections in the periodontal tissues. This immunosuppression allows pathogenic bacteria to thrive and dominate, further driving the dysbiotic state.^[39]^ Besides, high glucose levels in the gingival crevicular fluid of diabetic patients can serve as a nutrient source for pathogenic bacteria, thus promoting their overgrowth and the displacement of commensal species, leading to dysbiosis.^[40]^ This change is characterized by an increased abundance of periodontopathogens, which are more adept at surviving in a sugar-rich environment and can outcompete commensal bacteria.^[41]^ Periodontopathogens, including P.g, *Tannerella forsythia*, and *Treponema denticola*, collectively referred to as the “red complex,” demonstrate an increased prevalence. These bacterial species are equipped with distinct virulence factors, facilitating their evasion of host immune responses and consequent disruption of tissue homeostasis. This ability to circumvent host defenses and alter the equilibrium of the periodontal microenvironment underscores their critical role in periodontal disease pathogenesis.^[4]^ The imbalance in the microbiome can increase the susceptibility to periodontal disease as periodontopathogens become more prevalent. For example, P.g can alter the oral microbiome by promoting a more permissive environment for other pathogens, which contributes to the progression of periodontal disease.^[42]^ The inflammatory status caused by P.g can prime the periodontal tissues for an exaggerated response to bacterial plaque.^[43]^ Chlorhexidine has been extensively studied and used as an adjunctive treatment for periodontal therapy due to its antimicrobial properties. Its efficacy in controlling periodontal inflammatory dysbiosis in diabetic patients is rooted in its ability to reduce microbial load and oral biofilm formation, which are key elements in periodontal disease progression.

## 3. Pharmacotherapeutics of chlorhexidine

Chlorhexidine’s antimicrobial effectiveness varies with concentration, being bacteriostatic at lower levels (0.02%–0.06%) and bactericidal at higher ones (>0.12%). While effective against many microbes, its efficacy is less against certain fungi, mycobacteria, and viruses. Its activity can be diminished by organic matter or negatively interacting substances like soap or anionic compounds.^[44]^

### 3.1. Antimicrobial effects

**Figure 2.**
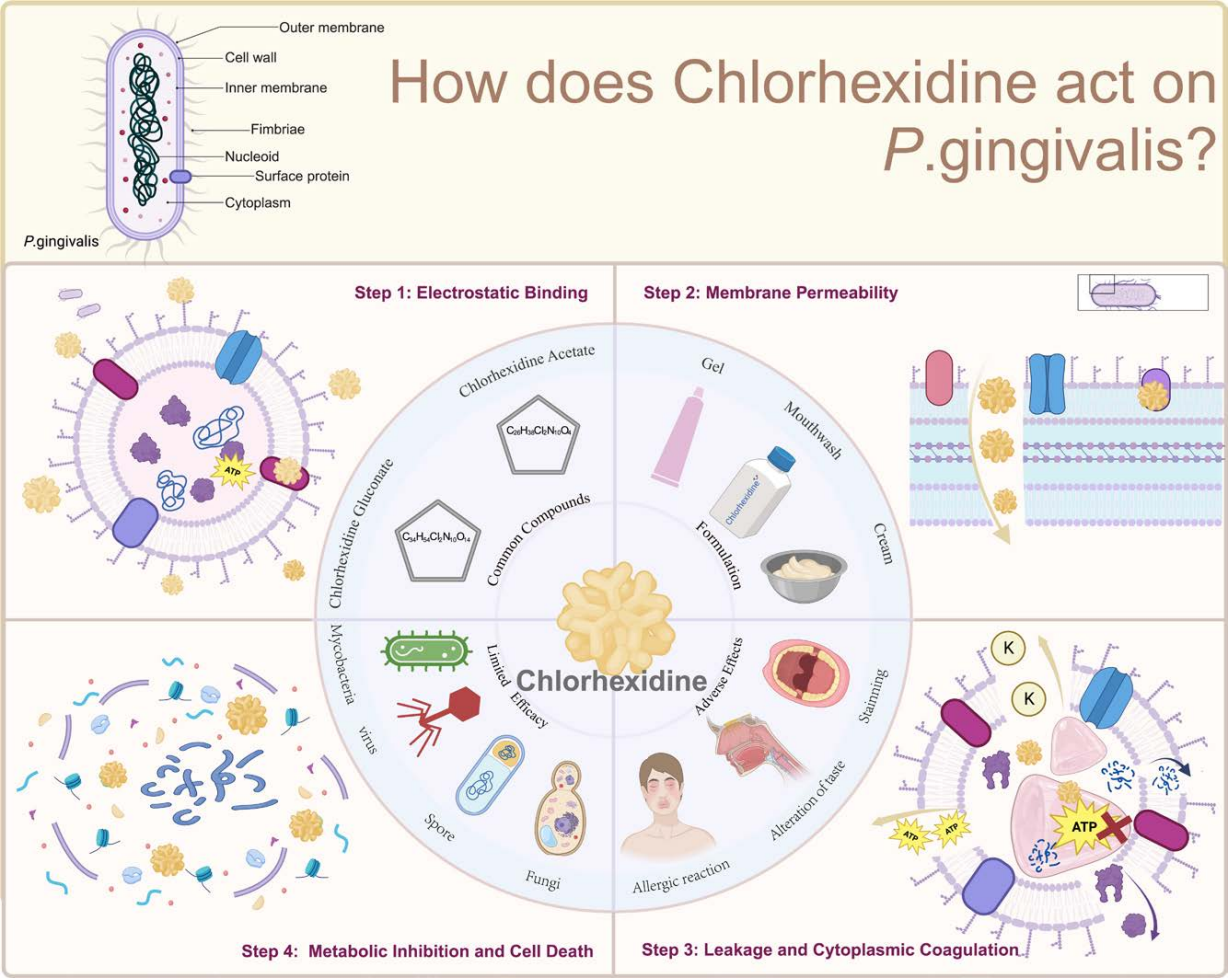
The mode of action of chlorhexidine on *Porphyromonas gingivalis*. Created with BioRender.com.

Chlorhexidine is an antiseptic that is commonly used in oral health care products because of its broad-spectrum antibacterial activity. It works against a variety of bacteria, including P.g, which is a key pathogen implicated in periodontal disease (shown in Figure 2). The mode of action of chlorhexidine on P.g is as follow:

- Initial contact binding: Chlorhexidine is a cationic (positively charged) molecule that is attracted to the anionic (negatively charged) components of the microbial cell wall and membrane. The chlorhexidine molecule attaches itself to the cell wall and membrane phospholipids.^[45]^
- Membrane disruption: After binding, chlorhexidine disrupts the integrity of the microbial cell membrane. This increases the membrane’s permeability, making it leaky.^[46]^
- Leakage and coagulation of cytoplasm: Essential ions and molecules inside the cell start to leak out due to the compromised membrane. Chlorhexidine enters the microbial cell due to the increased permeability and causes coagulation of the cytoplasmic contents, which impairs cellular function.
- Metabolic inhibition and cell death: The coagulation of the cytoplasm interferes with vital cellular processes, including metabolism and DNA/RNA synthesis. Eventually, the cells can no longer maintain their integrity, leading to more substantial leakage and cell death.^[47]^

### 3.2. Antiplaque effects

**Figure 3.**
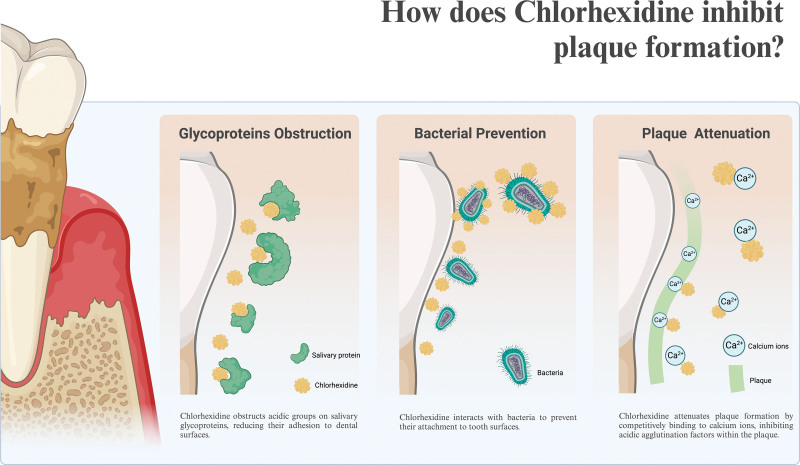
The antiplaque effect of chlorhexidine. Created with BioRender.com.

Pharmacokinetic studies of oral chlorhexidine rinses indicate that approximately 30% of the active ingredient is retained in the mouth following rinsing, which is subsequently slowly released into oral fluids.^[48]^ This ability to adsorb to dentin, shared with tetracycline antibiotics such as doxycycline, is known as “substantivity” and is the result of chlorhexidine’s positive charge. It is likely that this substantivity plays at least some role in chlorhexidine’s antimicrobial activity, as its persistence on surfaces such as dentin prevents microbial colonization. The mechanisms of plaque inhibition by chlorhexidine are as follows (shown in Figure 3):

- Glycoprotein obstruction: The blocking of acidic groups on salivary glycoproteins reduces protein adherence to tooth surfaces.
- Bacterial prevention: Binding sublethal amounts of certain substances to bacteria in salivary coats, including their polysaccharide layers, may disrupt their ability to adhere to teeth. This suggests a potential strategy for preventing bacterial colonization on dental surfaces.
- Plaque attenuation: By precipitating the acidic agglutination factors in saliva and displacing calcium, which is involved in “gluing” plaque together.

Chlorhexidine plays a valuable role in dentistry and antiseptics. Studies have confirmed its beneficial effects in reducing plaque accumulation, preventing tooth decay, managing gingivitis, treating periodontitis, and addressing alveolar osteitis. However, its use should be under the guidance of a dental professional, given the potential side effects such as mucosal irritation and staining when used over a prolonged period, and its effectiveness depends on the pH of the environment and the presence of organic substances.^[49]^

## 4. Analysis of the impact of chlorhexidine in patients with DM

### 4.1. Impact of chlorhexidine on periodontal parameters and pathogens

Studies have consistently shown that chlorhexidine, when used as an adjunct to mechanical debridement, can significantly reduce dental plaque, which is a key etiological agent in the development of periodontal diseases.^[50]^

Periodontopathic bacterial species in the oral cavity are categorized according to their virulence. Species belonging to the red complex, including P.g, *T denticola*, and *T forsythia*, are highly virulent and play a significant role in the progression of periodontitis.^[51]^ Numerous in vitro studies have demonstrated that 0.01% to 0.2% chlorhexidine glucoronate has a potent bactericidal effect on single species and multispecies cultures containing *Streptococcus mitis*, *Fusobacterium nucleatum*, *Porphrymonas gingivalis*, and *Aggregatibacter actinomycetemcomitans*.^[42,52]^ Matayoshi found that after type 2 diabetes patients used chlorhexidine gluconate mouthwash for six months, there was a significant reduction in the quantity of red complex bacteria in their saliva, and their blood HbA1c levels were significantly lowered.^[53]^ Almeida et al conducted a 14-day treatment involving chlorhexidine application and irrigation in the periodontal pockets of diabetic patients. They observed improvements in probing depth and a significant reduction in *T forsythia* levels, and the effects lasted for up to a year.^[54]^ Overall, studies indicate that the antimicrobial action of chlorhexidine not only improves periodontal health by reducing plaque and bacterial levels but also shows beneficial effects on glycemic control in diabetic patients (Table 1).

**Table 1:** Included treatment studies reporting study characteristics, clinical parameters, results, and quality assessment.

Authors, year of publication	Study method	Number of included patients	Study duration	Chlorhexidine concentration and type	Clinical parameters	Study results	Quality*
Santos et al, 2013^[55]^	Randomized controlled clinical trial	38	12 mo	0.12% chlorhexidine solution and 1% chlorhexidine gel	Glycated hemoglobin, fasting plasma glucose, and CAL	No significant differences in clinical parameters or glycemic condition between groups	+
Faramarzi et al, 2017^[56]^	Randomized controlled clinical trial	68	6 mo	Xanthan-based 1.5% CHX gel	Fasting blood sugar, HbA1c, and CAL	Significant reduction in fasting blood sugar and HbA1c, improvement in clinical attachment levels	+
Lipski et al, 2021^[57]^	Cohortl study	42	6 mo	0.20% chlorhexidine toothpaste and mouthwash	Oxidative stress markers in saliva, HbA1c, CRP, and GI	Reduction in thiobarbituric acid in saliva and improvement of periodontal status	±
Engebretson et al, 2013^[58]^	Randomized clinical trial	514	6 mo	0.12% chlorhexidine oral rinse	PPD, CAL, BOP, GI, and fasting glucose	No significant difference in HbA1c levels between groups, improvement in periodontal measures	+
Srirangarajan et al, 2016^[59]^	Randomized controlled clinical trial	60	6 mo	1% chlorhexidine gel 0.2% chlorhexidine solution	PI, GI, PPD, FG, insulin, C-reactive, CRP, and HOMA-IR	A significant reduction in PI, PPD, FG, HOMA-IR. The CRP levels were consistent compared to baseline	+
Marconcini et al, 2021^[60]^	Randomized controlled clinical trial	60	6 mo	Chlorhexidine toothpaste (0.20%) and chlorhexidine mouthwash (0.20%)	HbA1c, PPD, PI, BOP, ROMs, and glycated hemoglobin	Significant reduction in plasma ROM and improvement of periodontal parameters	+
Schara et al, 2006^[61]^	Pilot trial	10	12 mo	Not specified	HbA1c, PI, BOP, PPD, and CALs	A significant reduction in the serum level of HbA1c within 3 mo	±

* + = good quality, ± = doubtful quality, BOP = bleeding on probing, CAL = clinical attachment level, CRP = C-reactive protein, FG = fasting glucose, GI = gingival index, HOMA-IR = homeostasis model assessment of insulin resistance, PPD = probing pocket depth, ROMs = reactive oxygen metabolites.

### 4.2. Impact of chlorhexidine on glycemic control and insulin resistance

There’s emerging evidence suggesting that successful management of periodontal diseases with chlorhexidine can positively influence glycemic control, which is critical for diabetic patients. Improved periodontal health may reduce systemic inflammation and insulin resistance, thus aiding in better management of diabetes.^[62]^ In a study involving 68 participants with advanced periodontitis and glycated hemoglobin levels above 6%, those treated with periodontal therapy that included chlorhexidine gel along with SRP showed greater reductions in fasting blood glucose and HbA1c compared to those who received only SRP.^[63]^ Schara concluded that full-mouth disinfection using chlorhexidine mouthwash can significantly improve periodontal health and metabolic control in patients with type 1 diabetes who suffer from periodontitis.^[56]^ However, in a randomized controlled trial with 514 participants, Engebretson et al^[61]^ found that nonsurgical periodontal treatments, including chlorhexidine, did not improve HbA1c levels in patients with type 2 diabetes and moderate to advanced chronic periodontitis. Santos found that 12 months after periodontal disinfection with chlorhexidine, there were no significant improvements in clinical periodontal parameters, glycated hemoglobin, or fasting blood glucose levels in type 2 diabetes participants.^[58]^

In addition to clinical parameters such as probing depth and attachment loss, improvement in insulin sensitivity is needed for diabetes patients. A study demonstrated that full-mouth disinfection with 0.2% chlorhexidine significantly reduced the homeostasis model assessment of insulin resistance index in diabetic patients, indicating its effectiveness in improving insulin resistance.^[55]^ However, a systematic review concluded that the evidence supporting a causal relationship between nonsurgical periodontal treatments, including full-mouth disinfection, and insulin resistance is weak and contradictory.^[59]^

### 4.3. Impact of chlorhexidine on oxidative stress

The anti-inflammatory effect of chlorhexidine can help reduce the levels of proinflammatory cytokines in the gingival crevicular fluid, substances that are known to be higher in diabetics and associated with poor periodontal outcomes.^[64]^ Inflammation is considered a key mediator between periodontitis and diabetes, and the impact of chlorhexidine on oxidative stress markers has also garnered attention. Studies indicate that the use of digluconate chlorhexidine can reduce oxidative stress markers in the saliva of type 1 diabetes patients, such as advanced oxidation protein products and thiobarbituric acid.^[63]^ Furthermore, a study evaluating the effectiveness of nonsurgical periodontal treatment in diabetic patients through the measurement of oxidative stress outcomes found that periodontal therapy, including the use of chlorhexidine mouthwash, helps in reducing reactive oxidative metabolites.^[57]^

### 4.4. Adverse effects and limitation of chlorhexidine in diabetic patients

The use of chlorhexidine mouthwash can lead to a range of side effects. McCoy reported that out of 140 diabetic patients treated with chlorhexidine gluconate mouthwash for periodontal therapy, 44 (31%) experienced adverse events, including tooth staining and loss of taste.^[60]^ Chlorhexidine’s antimicrobial effects are recognized for their potential to reverse microbial dysbiosis. However, its nonspecific antibacterial activity can also inhibit beneficial oral bacteria, including species such as Veillonella, Actinomyces, Haemophilus, Rothia, and Neisseria.^[65]^ These bacteria play a crucial role in reducing dietary nitrates to nitrites in the saliva, a process that contributes to cardiovascular health through the release of nitric oxide.^[66]^ Joshipura et al suggested that chlorhexidine mouthwash can reduce the production of oral nitric oxide and plasma nitrate levels in healthy subjects, potentially leading to an increase in blood pressure.^[67]^ Additionally, studies indicate that mouthwash may increase the risk of developing prediabetes/diabetes within 3 years by impairing the oral bacteria’s ability to reduce nitrates.^[68]^ Consequently, the use of chlorhexidine mouthwash might negate the beneficial effects of a nitrate-rich diet mediated by the oral microbiome.^[69]^ Another emerging concern regarding the use of chlorhexidine is the development of antimicrobial resistance, which represents a significant adverse effect.^[70]^ The risk of chlorhexidine resistance in oral bacteria and potential cross-resistance to antibiotics remains poorly understood. Thus, future studies should concentrate on investigating the effects of chlorhexidine on bacteria within oral biofilms. It is worth noting that there is currently no literature providing evidence for the use of chlorhexidine in periodontal treatment for patients with gestational diabetes. However, a meta-analysis, assessing potential heterogeneity of chlorhexidine use, has demonstrated that chlorhexidine mouthwash combined with SRP can effectively reduce the risks of preterm birth and low birth weight.^[17]^ Further research is needed to establish the safety of chlorhexidine for periodontal treatment in patients with gestational diabetes.

### 4.5. Treatment strategy integrating periodontology and internal medicine

A diversified treatment strategy that integrates chlorhexidine treatment with comprehensive diabetes management to manage periodontitis relapse might be advocated. Regular dental checkups and professional periodontal treatment to monitor and manage oral health status are needed.^[71]^ Collaboration with the patient’s endocrinologist for comprehensive management of diabetes and periodontal disease is essential, involving tailored diet, exercise, and medication plans to improve overall systemic and oral health outcomes.^[72]^ Personalized diabetes education and lifestyle counseling to help patients improve their dietary habits and increase physical activity, thereby achieving better glycemic control. Individualized oral hygiene education is crucial, highlighting the importance of proper brushing and flossing techniques alongside regular professional dental cleanings to maintain optimal oral health.^[73]^ Through this proactive and comprehensive treatment approach, we can more effectively manage the periodontal health of diabetic patients, thus contributing to optimal glycemic control and improved quality of life.

## 5. Conclusion and future objectives

This review highlights the utility of chlorhexidine as an adjunct treatment in the management of periodontitis in patients with DM. Its broad-spectrum antimicrobial properties are crucial for controlling oral biofilms and periodontopathogens, effectively reducing periodontal pocket depth and inflammation. However, the use of chlorhexidine is not devoid of side effects such as discoloration, altered taste, and potential antibiotic resistance, which necessitates cautious use and consideration of alternative therapies. Future research should focus on the long-term effects and safety of chlorhexidine across different diabetic populations, including those with gestational diabetes, and delve deeper into the systemic benefits of improved periodontal health, such as enhanced glycemic control and insulin resistance. Moreover, close collaboration between dentistry and endocrinology is essential to optimize periodontal treatment in diabetic patients, ensuring that it complements broader metabolic management strategies. This interdisciplinary approach is crucial for developing comprehensive treatment plans that improve both systemic and oral health outcomes.

## Author contributions

**Writing – original draft:** Honglan Sun, Shizhao Chen, Chao Yang.

**Investigation:** Chao Yang, Yuqi Huang.

**Formal analysis:** Huifang Kuang, Yuqi Huang.

**Methodology:** Huifang Kuang.

**Conceptualization:** Xiaoning He, Wen Luo.

**Writing – review & editing:** Xiaoning He, Wen Luo.
